# Advances in lymphatic metastasis of non-small cell lung cancer

**DOI:** 10.1186/s12964-024-01574-1

**Published:** 2024-04-02

**Authors:** Xiaofei Zhang, Li Ma, Man Xue, Yanning Sun, Zhaoxia Wang

**Affiliations:** Cancer Medical Center, The Second Affiliated Hospital of Nanjing Medical University, Nanjing, 210011 China

**Keywords:** NSCLC, Lymphatic metastasis, Lymphatic endothelial cells, Vascular endothelial growth factor, Immune microenvironment, Biomarker

## Abstract

Lung cancer is a deeply malignant tumor with high incidence and mortality. Despite the rapid development of diagnosis and treatment technology, abundant patients with lung cancer are still inevitably faced with recurrence and metastasis, contributing to death. Lymphatic metastasis is the first step of distant metastasis and an important prognostic indicator of non-small cell lung cancer. Tumor-induced lymphangiogenesis is involved in the construction of the tumor microenvironment, except promoting malignant proliferation and metastasis of tumor cells, it also plays a crucial role in individual response to treatment, especially immunotherapy. Thus, this article reviews the current research status of lymphatic metastasis in non-small cell lung cancer, in order to provide some insights for the basic research and clinical and translational application in this field.

## Introduction

Lung cancer ranks as the leading cause of cancer-related deaths [1]. Non-small cell lung cancer (NSCLC) accounts for approximately 85% lung cancer [2]. Despite the advanced medical treatments now available, the long-term prognosis is still disillusionary. Patients who underwent surgery at an early stage are still susceptible to metastasis or recurrence [3, 4]. In NSCLC, lymph node (LN) metastasis is the most primary and crucial metastatic route [5, 6]. Clinical treatment and prognosis also depend on the extent of local LN involvement, known as ‘N-stage‘ [7, 8]. This may be due to the presence of occult micrometastatic cancer cells prior to diagnosis and surgery, which include LN micrometastasis [4, 9].

**Fig. 1:**
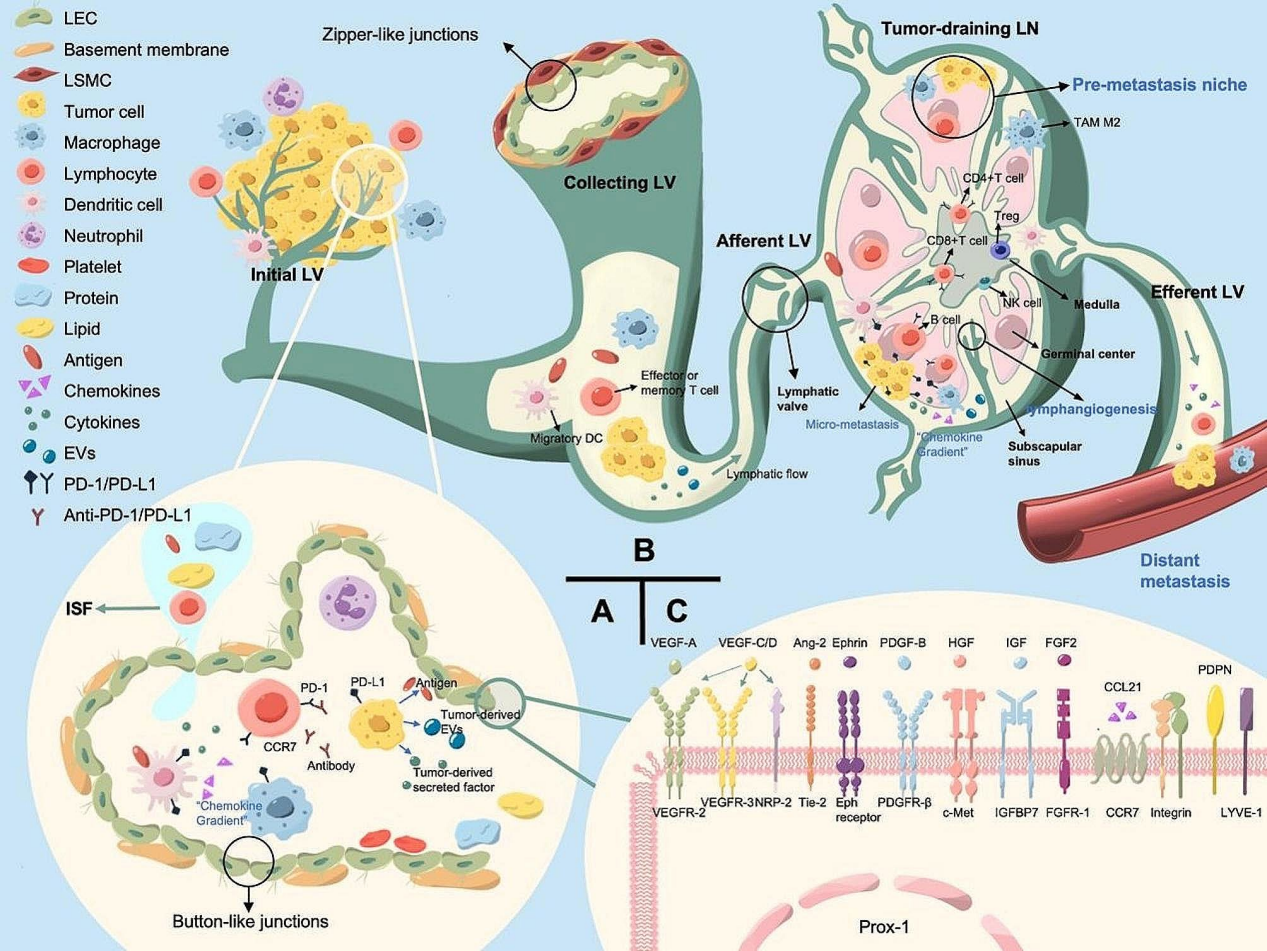
The structure of lymphatic system and tumor-associated lymphatic metastasis in NSCLC. (A) Initial lymphatic vessel (LV) is lined by a single layer of LECs without a continuous basement membrane to uptake ISF and macromolecules by the blood vessels. LECs produce the chemokine gradients of CCL21 to entry immune cells expressing CCR7 into initial LV. (B) The lymphatic flow reaches TDLN though afferent LV, where the lymph permeates in subcapsular and medullary sinuses and eventually come into the efferent LV. Tumor cells, leukocyte, migratory dendritic cells (DCs), antigens, tumor-derived secreted factors (TDSFs), and tumor-derived extracellular vesicles (EVs) drain the subcapsular sinuses (SCS). Newborn LECs create and maintain chemokine gradients that direct DC migration. And CD169 + SCS macrophages capture tumor-derived material for antigen presentation. These culminate a pre-micrometastasis niche that crosses metastasis and immunity supporting tumor cell proliferation, adhesion, invasion, and LN immune suppression. (C) Various cells, including tumor cells, secrete growth factors to activate receptors on the LEC surface to regulate LEC growth and migration and remodel LV networks. Lymphangiogenesis-mediating receptors involve ligands or interacting proteins, homologous receptors, and specific LECs markers

In human body, the two fundamental roles of lymphatic vessels are fluid transport and immunosurveillance [10]. Lymphatic vascular system can transport fluids, cells, and biomolecules between peripheral tissues and the circulatory system, maintaining tissue homeostasis [11]. Initial lymphatic vessels are lined by a single layer of lymphatic endothelial cells (LECs) in button-like junctions, with a discontinuous basement membrane that lacks smooth muscle cells [12]. Unidirectional and blunt-ended initial lymphatic capillaries uptake extravasated interstitial fluid (ISF) rich in leukocytes, lipids and proteins and form lymph, which is then transported into collecting lymphatic vessels. The endothelial cells of the collecting lymphatic vessels are connected in tighter zipper-like junctions and surrounded by smooth muscle cells, and have special valves prevented retrograde flow [13, 14] (Fig. 1). Lymph drains various antigens and activated antigen-presenting cells to LNs, while delivering immune cells and response factors back to bloodstream [12]. Lymphatic system can actively promotes tumor cells migrating to lymphatic vessels [15], meanwhile tumor-derived growth factors stimulate intratumoral and peritumoral lymphangiogenesis, facilitating lymphovascular invasion (LVI) to remodel tumor microenvironment (TME) [16]. Cancer cells that invade lymphatic vessels spread into tumor-draining lymph nodes (TDLN) to induce neo-lymphangiogenesis. Sprouting and proliferation of LECs mediate lymphatic expansion in tumor-draining LNs [17]. This constitutes an intermediate platform for lymphatic metastasis of cancer cells, formatting the lymphatic pre-metastasis ecological niche and creating a favorable microenvironment for metastasis [18](Figure.1). In recent years, immunotherapy has brought wish to patients with advanced NSCLC, while the immunotherapy effect of different populations seems to be different, and the expression of programmed death 1 (PD-1)/ programmed death ligand 1 (PD-L1) is the main drug reference index in clinical practice [19]. As an important component of TME, lymphatic system functions an important role in regulating immune response [20].We summarized some interesting genes, which regulated lymphatic metastasis of NSCLC, exerted synergistic role in immunotherapy at the same time. They seems to have great potential to be used as prognostic indicators for evaluating immunotherapy and synergistic therapeutic targets just like tumor anti-angiogenesis therapy [21, 22]. Lymphatic metastasis have been reported in other carcinomas, such as breast cancer [23], ovarian cancer [24], cervical cancer [25], prostate cancer [26], bladder cancer [27], liver cancer [28], gastric cancer [29], pancreatic cancer [30], colorectal cancer [31] and so on, however, the mechanism of lymphatic metastasis in NSCLC has not been systematically reviewed. Understanding the mechanism of tumor-associated lymphangiogenesis is of great significance in prognosis and therapeutic target identification in patients with NSCLC lymphatic metastasis.

**Table 1:** Clinicopathologic characteristics and prognosis in NSCLC lymphatic metastasis

Makers	Tumor simple size	Function	Related molecules and pathways	Clinicopathologic characteristics	Prognosis	Ref.
ANGPTL2	233	Accelerator	VEGF-A	More LNM and advanced TNM stage	Poorer OS	[62]
ANGPTL2	81	Accelerator	NF-ĸB	More TAM infiltration and larger tumor size	Poorer OS	[64]
Ang-2	575	Accelerator	-	More LNM and advanced tumor stage	Poorer OS	[65]
PDGF-B	109	Accelerator	PDGFR-β/ VEGF-C	More LNM, larger tumor size, and worse histological differentiation	Poorer OS	[68]
PDGF-A PDGF-B	335	Accelerator	VEGFR- 3	More LNM	Poorer DSS	[69]
PDGF-B	127	Accelerator	PDGFR-β	More LNM, LMVD, advanced TNM stage and higher PLT counts	-	[70]
Platelet	852	Accelerator	VEGF-C	More LNM	-	[71]
Platelet	883	Accelerator	-	More LNM and advanced TNM stage	Poorer OS	[72]
HGF-α	113	Accelerator	c-Met/VEGF-C	More LNM and LMVD	Poorer OS	[73]
IGFBP7	97	Accelerator	-	More LNM and ptLVD	-	[74]
FGF2	335	Accelerator	VEGFR-3	Higher performance status, advanced pathologic T-stage, N-stage, worse histologic differentiation, and more vascular infiltration	Poorer DSS	[75]
Shh signal pathway	40	Accelerator	LYVE-1	Worse histology and more severe visceral pleural invasion	Poorer OS	[76]
STAT3	50	Accelerator	-	More LN micrometastasis	-	[77]
SCC-S2	25	Accelerator	-	More LNM, TNM, and ki-67 expression	Poorer OS	[78]
SCP3	89	Accelerator	VEGF-C VEGF-D	-	Poorer OS	[79]
EV-packaged circTLCD4-RWDD3	312	Accelerator	PROX1	More LNM and MLVD	Poorer OS and DFS	[83]
MiR-128	30	Inhibitor	VEGF-C/ VEGFR-3/ERK/AKT/p38	Less LNM, lower pathological stage, and better differentiation	-	[85]
MiR-22	49	Inhibitor	VEGFR-3	Less LNM, MLVD, lower TNM stage, smaller tumor size, and less vascular invasion	-	[86]
CCR7	90	Accelerator	VEGF-D	More LNM, LVD, and advanced TNM stage	Poorer OS	[49]
IL-7/IL-7R	100	Accelerator	VEGF-D	More LNM, LVD, and advanced TNM stage	Poorer OS	[92]
IL-17	52	Accelerator	LYVE-1+	More LVD and advanced TNM stage	Poorer OS and DFS	[94]
IL-17	36	Accelerator	VEGF-C	More LNM, LVD, and advanced tumor grade	-	[93]
COX-2	65	Accelerator	VEGF-C	More LNM and LMVD	-	[97]
Romo1	30	Accelerator	ROS/VEGF-C	-	Poorer OS and DFS	[98]
CD147	46	Inhibitor	TGF-β/CD147 methylation	Less LNM, TNM, and smaller tumor size	-	[119]
KAI1	312	Inhibitor	β-catenin/EMT	Less LNM, LVD, and MVD	Better OS and DSS	[121]
MiR-148a	48	Inhibitor	DNMT1/E-Cadherin/	Less LNM and lower clinical stage	Better OS and DFS	[122]
SNAI2 TWIST1	160	Accelerator	EMT	-	Poorer OS and RFS	[125]
JAM-C	140	Accelerator	ERK/VEGF-C	More LNM and faster tumor growth	Poorer OS and RFS	[48]
p-S6	350	Accelerator	-	More LNM and worse histological type	Poorer OS	[127]

**Table 2:** Molecular mechanisms and main signaling pathways of NSCLC lymphatic metastasis

Makers	Function	Related molecules and pathways	Cell lines	Phenotype	Animal studies	Ref.
Mdig	Inhibitor	HIF-1α/VEGF-C /VEGF-/D/VEGFR-3	A549, H1299, 293T, HUVECs, HLECs	-	Female athymic nu/nu mice	[52]
C/EBP-δ	Accelerator	HIF-1α/VEGF-C/VEGFR- 3	Human HMVEC-LLy, Lewis lung adenocarcinoma cells (3LL)	Migration, vascular network formation, and apoptosis of LECs	C57BL/6J mice and C/EBP-d-null mice	[53]
EFNA4	Accelerator	Ephrin A4	H1299, A549, PC9	Proliferation, migration, and adhesion	Male BALB/c nude mice	[57]
ANGPTL2	Accelerator	HIF-1/ VEGF-A	H1299, A549	-	-	[62]
ANGPTL2	Accelerator	integrin α5β1/ VEGF-A/p38/NF-κB	A549, CL1-0, CL1-5	Migration and tube formation of LECs	BALB/c nude mice	[63]
ANGPTL2	Accelerator	NF-κB/ TAMs M2 polarization	H1299, A549, HUVECs	Proliferation, invasion, and migration LEC tube formation	BALB/c nude mice	[64]
NRP-2	Accelerator	TGF-β/VEGF-C/ HGF/c-MET/ GIPC1/PTEN	H358, A549	Migration, invasion, EMT, and EGFR resistance	NU/J (Foxn1nu/nu) mice	[55]
IGFBP7	Accelerator	-	LLC, L929	LEC tube formation	Female C57BL/6 mice and BALB/c nude mice	[74]
Shh signal pathway	Accelerator	Shh/ Gli1/LYVE-1	H1299, H2009, Calu-1	Proliferation	-	[76]
circTLCD4-RWDD3	Accelerator	UBC9/ SUMOylated hnRNPA2B1/ALIX/ESCRT-III/PROX1	A549, H1299	Migration and tube formation of HLECs	BALB/c nude mice	[83]
MiR-128	Inhibitor	VEGF-C/ VEGFR-3/ERK/AKT/p38	A549, SK-MES-1, NCI-H460, HUVECs	Proliferation, migration, invasion, apoptosis, and angiogenesis	BALB/c nude mice	[85]
CXCR4 CCR7	Accelerator	VEGF-C/ VEGFR-2/VEGFR-3/ERK/p38/ AKT	A549	Proliferation and invasion	BALB/c nude mice	[88]
CCR7	Accelerator	VEGF-D/ERK/AKT	BE1, A549	-	-	[49]
CCR7-CCL21	Accelerator	TNF-α/NF-κB/VEGF-D	A549, H460	Invasion and metastasis	-	[90]
IL-7/IL-7R	Accelerator	c-Fos/c-Jun/VEGF-D	A549, SPC-A1, H460, LH7, SK-MES-1	Migration, invasion, and tumorsphere formation	-	[92]
IL-17	Accelerator	VEGF-C/ ERK	LLC, A549, SPC-A-1	Tube formation and chemotaxis of LECs	-	[93]
IL-1α	Accelerator	CXC chemokines/IKKb/NF-kB/VEGF-C	LNM35, N15, LLC/IL-1b	M2-like TAMs migration	Male KSN/slc mice	[95]
COX-2	Accelerator	PGE2/ VEGF-C/ EP1/4 receptors	Anip973, AGZY83-a	-	BALB/c nude mice	[96]
TSLP	Accelerator	LSP/IL-4/IL-13/VEGF-C	HLM, Monocyte, monocyte-derived macrophage	-	-	[99]
LEDGF	Accelerator	STRE/VEGF-C	A549, H1299	-	Female CD-1 nude mice	[100]
TGF-β1	Accelerator	VEGF-C	A549, NCI-H1993, NCI-H358	Proliferation, invasion, EMT, and CSCs	-	[118]
TGF-β1	Accelerator	-	H157, HMVEC-LLy	Adhesion and transmigration	Female athymic nude mice	[126]
CD147 methylation	Inhibitor	TGF-β/KLF6/MeCP2/DNMT3A/Sp1/Tet1/TDG/SMAD2/3	A549, NCI-H226, NCI-H460	Proliferation, invasion, and migration	Female BALB/c nude mice	[119]
Cathepsin heparanase	Accelerator	VEGF-C /VEGFR-3	Macrophage, LLC, 4T1, A549, LM2-4	Migration, invasion, and paclitaxel resistance	-	[115]
miR-148a	Inhibitor	DNMT1/E-Cadherin/	A549, H1299	Migration and invasion	-	[122]
JAM-C	Accelerator	VEGF-C/β1 integrin/ERK	Anip973, AGZY83-a	Migration	BALB/c nude mice	[48]
p-S6	Accelerator	-	A549, SPC-A1	Migration and invasion	-	[127]
sVEGFR-2	Inhibitor	VEGF-C/VEGFR-2/VEGFR-3/MMP	LLC	-	C57BL/6 mice	[154]
EGFR-TKIs	Inhibitor	VEGF-C/VEGFR-2/ VEGFR-3/CCR7/JAK/STAT3/ c-Myc	HCC827, HLECs	Proliferation, migration, and lymphatic tube formation of LECs	Nude mice	[155]
Nintedanib	Inhibitor	VEGF-C	H1993	-	NOD/SCID mice	[156]
Anakinra	Inhibitor	IL-1α/VEGF-C	LNM35, N15, LLC/IL-1b	CXC chemokines and M2-like TAMs migration	Male KSN/slc mice	[95]
HN-N07	Inhibitor	BIRC5/HIF1A/FLT4	-	-	-	[157]
FR -sema3C	Inhibitor	VEGF-C/ERK/AKT	HUVECs, HLECs, LM2-4, HEK293	Collapse of the LECs Cytoskeleton	-	[159]
Itraconazole	Inhibitor	VEGF-C	Endothelial cell	MPE	-	[160]

## Lymphatic vascular markers

During embryonic development, the formation of lymphatic vessels occurs after that of blood vessels. Venous endothelial cells express high levels of vascular endothelial growth factor receptor (VEGFR) -3 and upregulate LYVE-1 expression. The transcription factor SOX-18 interacts with the venous nuclear receptor COUP-TFII to express the lymphatic vessel specific TF Prox-1. Prox-1 in turn activates vascular endothelial growth factor (VEGF) -C-mediated VEGFR-3 expression to form stable lymphatic endothelial progenitor cells [15]. Subsequently, LECs express co-receptor Neuropilin-2 (NRP-2) synergistically sensitizing VEGF-C signaling. Under a VEGF-C concentration gradient, LECs express other specific markers, such as the transmembrane glycoprotein podoplanin(PDPN) and adhesion molecules, separating venous and lymphatic vessels [32, 33]. It was testified that LEC-specific marker genes expressed robustly in sequencing samples and distinct gene expression profiles in different LN LECs were modified by a similar pattern [17].

Except happening during embryonic development, the growth of new lymphatic vessels is induced in pathological circumstance.

Lymphatic vascular density (LVD) is used as an evaluation index for lymphangiogenesis [34], in addition, various LECs-specific endothelial markers have been used to evaluate tumor-associated lymphangiogenesis by immunohistochemical methods, including Prox-1 [34], VEGFR-3 [35, 36], LVYE-1 [37], Podoplanin [34], D2-40 [38, 39], NRP-2 [40] and others (Fig. 1). Numerous studies have shown the expression of VEGF-C/-D and VEGFR-3 positively correlated with LVI, LVD, and lymph node metastasis (LNM) in NSCLC [41, 42]. Peritumor had higher VEGF-C and VEGF-D expression and were associated with more advanced regional LNM [35]. In lung adenocarcinoma (LUAD), LVD is higher in the tumor stroma of PNPD + tissue [34], while in lung squamous cell carcinoma (LSCC), podoplanin does not promote cell migration, but also down-regulates VEGF-C by regulating JNK pathway [35]. Many markers affecting LNM in NSCLC were also associated with clinicopathological factors and prognosis (Table 1).

## Main signaling factors and their receptors of lymphangiogenesis in NSCLC

**Fig. 2:**
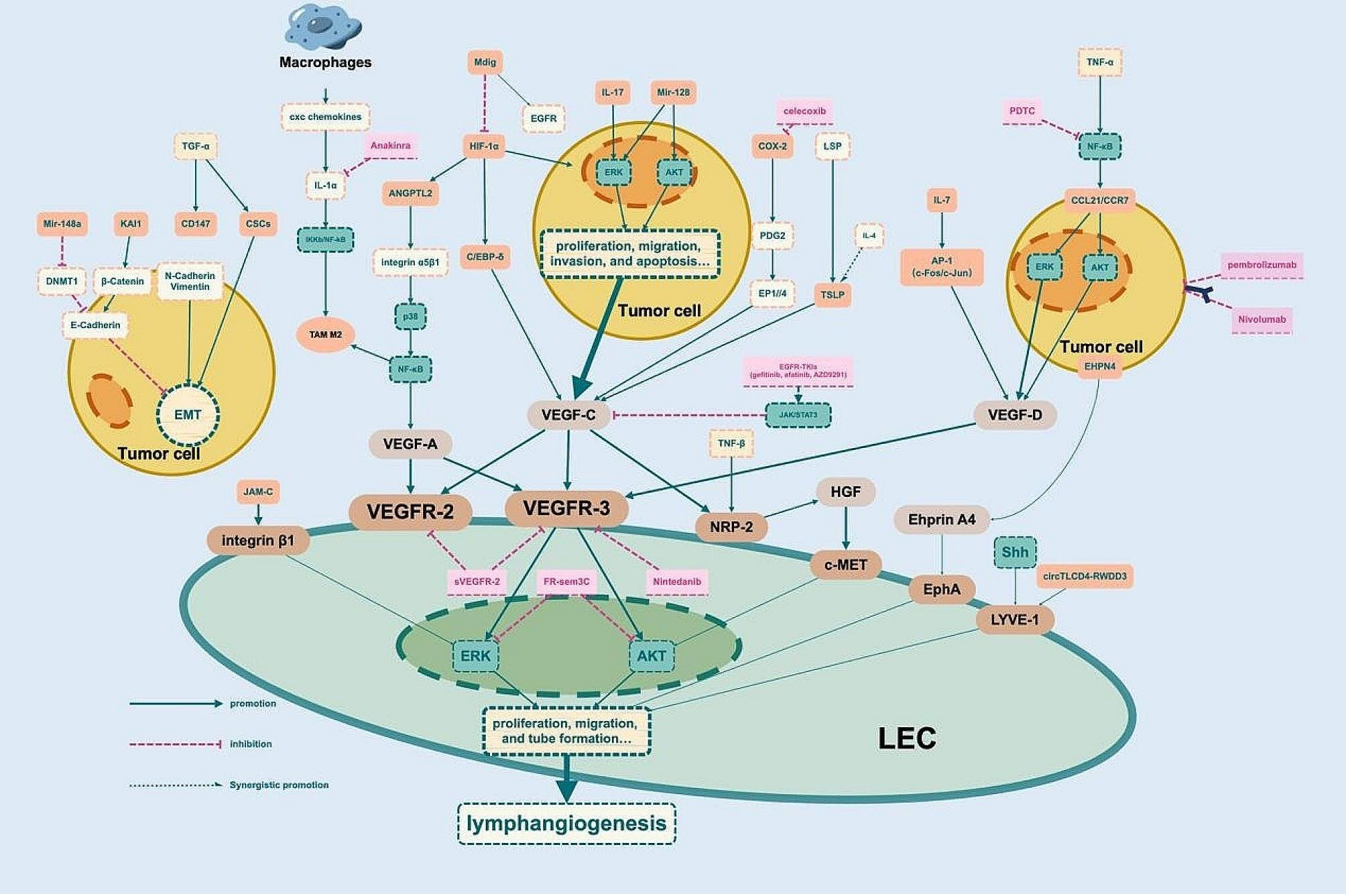
Main signaling pathways and major therapeutic targets contributing to lymphangiogenesis and lymphatic metastasis in NSCLC. In NSCLC, tumor cells induce the secretion of various factors that promote LECs-mediated lymphangiogenesis. Meanwhile this process contributes to the acquisition of the aggressive phenotypes, such as proliferation, migration, invasion, EMT, drug-resistance, and apoptosis in cancer cells

The central to lymphangiogenesis is the proliferation and migration of LECs. In NSCLC, LECs mediate lymphatic vessel formation and meanwhile contribute to the acquisition of a metastatic phenotype by cancer cells. Various secretory factors are induced to express and promote LECs-mediated lymphangiogenesis and lymphatic metastasis (Fig. 2; Table 2).

### VEGF

VEGF family belongs to the platelet-derived growth factor supergene family, which has a vital function in numerous physiological and pathological processes, especially angiogenesis and lymphangiogenesis. It encompasses seven homodimeric proteins, namely VEGF-A, VEGF-B, VEGF-C, VEGF-D, VEGF-E (virally encoded), VEGF-F (Snake venom VEGF) and placental growth factor (PIGF) [43, 44]. In the past we have focused a great deal of attention on the role of VEGF-A in regulating angiogenesis due to its important role in tumor homeostasis and metastasis [45]. Although VEGF-A also plays a role in tumor lymphangiogenesis, the VEGF-C/D/VEGFR-3 axis is currently recognized as a central molecular mechanism and major driver of lymphangiogenesis [46, 47]. VEGF-C and VEGF-D can be derived from various cells, including tumor cells, immune cells, etc [12]. VEGF-C and VEGF-D are upregulated in NSCLC and interact with VEGFR-3 to activate the MAPK/ERK or PI3K/AKT pathways, to stimulate LECs proliferation, migration and tube formation [48, 49]. Only lymphangiogenesis makes use of VEGFR-3 activation by VEGF-C/-D. Angiogenesis does not require this activation. VEGF-C has a substantially higher affinity for VEGFR-3, even though it can also bind to the VEGFR-2 on blood vascular endothelium in mature form. VEGFR-3 may also have a function in blood vessel formation by regulating VEGFR-2-mediated signaling [50].

In some studies, no angiogenesis was induced in the lesions despite growing LNs showing hypoxic markers [51]. Mineral dust-induced gene (Mdig), an oxygen-sensitive protein, might boost tumor growth and angiogenesis through activation of EGFR/VEGF-A/VEGF-R1/R2 pathway, it impeded lymphangiogenesis by blocking the HIF-1α/VEGF-C/D/VEGFR-3 axis [52]. Upon hypoxia, HIF-1α induced CCAAT/enhancer binding protein-δ (C/EBP-δ) to stabilize HIF-1α, forming a positive feedback mechanism. C/EBP-δ could regulate VEGF-C autocrine signaling through HIF-1α to promoted the transport of LECs and lymphatic vessel network generation in vitro [53].

NRP-2, a membrane coreceptor, was a significant molecular regulator of VEGFR-3 activation [54]. Transforming growth factor-β (TGF-β) signaling preferentially increased the abundance of NRP-2b, enhancing cellular migration, invasion, and tumorsphere formation in NSCLC [55]. When triggered by the corresponding ligand EphrinB2, the EphB4 receptor enhanced the internalization of VEGF-C/R-3 [56]. EFNA4, a member of the ephrin (EPH) family and encoded Ephrin A4 as the ligand for Eph receptors. It was upregulated in lung cancer patients with LNM and overexpression of EFNA4 contributed to the proliferation, migration and adhesion of lung tumor cells [57].

Some classical pathways of angiogenesis have been shown to play an important role in mediating lymphangiogenesis. As well, VEGF-A was proved to involve regulating and independently initiating lymphangiogenesis [58]. Higher expression of VEGF-A and VEGFR-3 has been proved associated with LNM in lung cancer cells [59], one study discovered the independence of VEGF-A-stimulated lymphangiogenesis, which is not exclusively dominated by VEGF-C/D [60]. The adipokine angiopoietin-like protein 2 (ANGPTL2) is an extracellular ligand for the angiopoietin receptor Tie2, its expression was upregulated in NSCLC with LNM and positively correlated with VEGF-A expression [61]. In hypoxic conditions, it has the potential to trigger the expression of HIF-1α and boost the abundance of both VEGF-A and ANGPTL2 [62]. In another study, ANGPTL2 was found to mediate VEGF-A-dependent LEC tube formation and migration through the integrin α5β1/p38/NF-κB pathway [63]. ANGPTL2 could also promote M2 polarization of tumor-associated macrophages (TAMs) through NF-κB pathway to enhance proliferation, invasion, migration of NSCLC cells and tube formation of LECs [64]. While VEGF-A appeared to correlate negatively with LNM in tumor mesenchyme, which might suggest the influence of tumor-mesenchymal interactions in lymphatic metastasis of NSCLC [59]. In addition, the formation of the lymphatic vasculature depends on the agonist role of Ang-2, and serum Ang-2 levels were associated with tumor dissemination and lymphatic invasion. The levels of Ang-2 were higher in patients with LNM in NSCLC [65, 66].

### Other growth factors

Platelet-derived growth factors (PDGFs) and their receptors, PDGFRs, are important factors affecting LNM in lung cancer [67]. PDGF-A was discovered to be paracrine by stimulating PDGFR-expressing LECs in tumor cells, co-expression of PDGF-B and VEGFR-3 in lung cancer tissues was connected with LNM and poor prognosis [68, 69]. Another study found that PDGF-B, PDGFR-β, and LMVD expression in tissues correlated with lymphatic metastasis, and was also highly correlated with elevated levels of platelet [70]. This implied that platelet secreted cytokines such as PDGFs to promote lymphangiogenesis. Meanwhile, platelet itself promoted tumor cell proliferation and metastasis. It prolonged the abnormal aggregation and survival of tumor cells in the peripheral circulation and guided in their immune escape. Studies found that elevated platelet counts (even when the counts were in the normal range) were significantly associated with higher rates of LNM. This mean that anti-platelet therapy might be a peculiar way to manage lymphangiogenesis and tumor progression in NSCLC patients [71, 72].

As well, the hepatocyte growth factors (HGFs) and its receptor c-Met were highly expressed in NSCLC. HGF-α/c-Met might synergize with the VEGF-C pathway in inducing lymphangiogenesis [73]. Noticeably, NRP-2b specifically promoted HGF-induced p-AKT, whereas inhibition of c-MET attenuated NRP-2b-dependent cells migration [55]. Insulin-like growth factor binding protein 7 (IGFBP7) expression was associated with metastatic clinicopathologic features and high LVD in NSCLC. It was also effective in enhancing LEC tubular structure formation and LNM [74]. Furthermore, the co-expressions of fibroblast growth factor 2 (FGF2) /VEGFR-3 and FGFR-1/PDGF-B in tumor cells were also significant predictors of a poor prognosis [75].

### Other mechanisms

As mentioned above, some genes might regulate growth factors such as VEGF-C through some pathways, but there were also other molecules and pathways that appear to be directly associated with NSCLC lymphangiogenesis. Shh and Gli1 in the sonic hedgehog (Shh) signaling pathway were significantly correlated with the expression of LYVE-1. Shh signaling pathway initiated LNM through LYVE-1-dependent lymphangiogenesis [76]. Overexpressed STAT3 was an independent LN micrometastasis risk factor and promoted micrometastasis in early-stage NSCLC [77]. Similarly, the expression of SCC-S2 was also linked with LNM in associated analysis [78]. Overexpression of SCP3 was found to be positively correlated with VEGF-C and VEGF-D and predicted poor prognosis in patients with LNM [79]. There was also a potential link between Syk and VEGF-C in LUAD [80], low Syk expression predicted a poorer independent prognosis [81, 82].

Moreover, an increasing number of non-coding RNAs have been demonstrated to regulate lymphangiogenesis. LECs absorbed EV-packaged circTLCD4-RWDD3, which stimulated Prox-1 transcription and induced lymphangiogenesis and LNM in NSCLC. The process of sorting of circTLCD4-RWDD3 into EVs was induced by SUMOylation of hnRNPA2B1, which could activate ALIX to recruit ESCRT-III [83]. In another way, it was identified the first LEC-specific long non-coding RNA, LETR1, could regulate critical targets involved in LEC survival and growth, which deserved to be explored in NSCLC in the future [84]. Similar effects were found in microRNA (miRNA/miR). MiR-128 were significantly downregulated in NSCLC tissues and cells, overexpressed MiR-128significantly curbed VEGF-C expression, which in turn restricted VEGFR-3-induced activation of ERK, p38, and AKT signaling pathways [85]. MiR-22 also negatively regulated VEGFR-3 to mediate lymphangiogenesis [86].

## Tumor cells-LECs-TME crosstalk in NSCLC lymphatic metastasis

### TME with chemokines and other cytokines

Patients in NSCLC with LNM have a stronger interplay network in the TME. Crosstalk between cancer cells and types of stromal cells in tumor tissue may lead to a specific metastasis microenvironment while contributing to LNM [87]. VEGF-C is a strong chemoattractant for macrophages and other inflammatory cells, which generate VEGF-C and influence the TME [54]. Tumor cells secrete other paracrine signaling molecules that function in parallel to VEGFR-3 to facilitate the recruitment of LVs. VEGF-C-mediated chemokines and their receptors are common factors mediating interactions in TME. When silencing VEGF-C, it had an inhibitory effect on the expression of VEGFR-2, VEGFR-3, CXCR4, and CCR7, and regulated tumor cells proliferation and invasion through AKT, ERK, and p38 signaling pathways [88]. CCR7 interacting with its ligands functioned in the directional migration of lymphocytes transporting and homing to LNs during tumor immune responses [89]. In metastatic lung cancer cell lines, overexpression of CCR7 greatly promoted VEGF-D expression and was regulated by the ERK1/2 and AKT signaling pathways [49]. In addition, TNF-α could activate NF-κB pathway in LECs to promote their secretion of CCL21 and promote metastasis and invasion of lung cancer cells through the CCR7-CCL21 axis. After specific antagonism of the NF-κB pathway with PDTC, CCL21 secretion was significantly reduced, and lung cancer cell metastasis was also reduced in the co-culture system of LECs and lung cancer cells [90].

So are the inflammatory factors, interleukin (IL)-7 induced the generation of c-Fos/c-Jun heterodimers through IL-7 receptor (IL-7R), which facilitated the binding of transcription factor AP-1 to the VEGF-D promoter [91, 92]. IL-17 upregulated VEGF-C levels in NSCLC cells through the p-ERK1/2 kinase pathway, which enhanced LECs chemotaxis and endothelial cord formation [93, 94]. IL-1α expression was enhanced and high levels of VEGF-A, VEGF-C, and VEGF-D were detected in macrophages in the tumor stroma. IL-1-driven inflammatory signaling induces CXC chemokines via cancer cells leading to M2-type macrophage recruitment [95]. The expression of COX-2 and VEGF-C levels was significantly increased in several highly metastatic lung cancer cell lines, COX-2-mediated VEGF-C expression dependent on the endogenous PGE2 pathway mediated by the EP1/4 receptor [96, 97]. Reactive oxygen modulator 1 (Romo1), which induced reactive oxygen species in mitochondria, affected cancer cell invasion and proliferation through sustained inflammation. Romo1 could induce lymphatic metastasis in NSCLC by modulating sustained inflammation/VEGF signaling [98]. Lipopolysaccharide (LSP) induced Thymic stromal lymphopoietin (TSLP) release from human lung macrophages (HLMs), which process could be enhanced by Th2 cytokine IL-4. Incubation of HLMs with TSLP induced the release of TNF-α, VEGF-A, angiopoietin 2 and VEGF-C, indicating the role of TSLP system in lymphangiogenesis through a Th2-dependent pathway in lung cancer and other chronic inflammatory disorders [99]. What’s more, contingency signals such as hyperthermia and oxidative stress stimulated the transcription factor LEDGF/p75 to activate VEGF-C signaling transcription, in order to control structural changes in the lymphovascular system [100]. In inflammatory environment, neutrophils could also increase the bioavailability and bioactivity of VEGF-A by secreting MMP-9 and heparanase, while secreting VEGF-D at a low extent to promote lymphangiogenesis [101].

### Immune microenvironment

TDLN is the specific site of tumor-induced tolerance. Primary tumors colonize LNs, which then influence the innate and adaptive immune system to advance the tumor [102]. LVs promote tumor metastasis and may increase anti-tumor effects by boosting tumor-associated antigens presentation to the immune system [103]. Immune surveillance occurs in the LNs by activating antigen-specific helper CD4 + T cells, cytotoxic CD8 + T cells and decoy receptors. Then it produces cytokines and other controlled immune responses to target cancer cells. Tumors induce immunosuppression by activating macrophages to help establish TME, promoting expression of suppressor molecules by cancer cells, and down-regulating cytotoxic T-lymphocytes (CTL). Metastatic LNs usually fail to construct effective anti-tumor immunity and can alter the sensitivity of cancer cells to immune surveillance [16, 104, 105].

High tumor mutational burden and PD-L1 expression in NSCLC were linked to enhanced intratumoral immune cell infiltration and LVI [106]. Tumor-infiltrating CD8 + T cells become dysfunctional and more immunosuppressive. PD-1 + CD + 8 T lymphocytes might be immune checkpoint blockade (ICB) -responsive and essential for anti-tumor immunity [107–109]. Moreover, host regulatory T cells (Treg) could induce conversion of naive tumor antigen-specific CD4 + T cells into anergy and peripherally induced Tregs in a genetically engineered lung adenocarcinoma mouse model. By specifically targeting Tregs, antitumor immune responses could be further enhanced by reducing the production of anergic T cells and releasing the brake on effector immunological response [110]. In metastatic LNs of primary NSCLC patients, PD-L1 + CSCs modulated the immune system by influencing affecting T-cell frequency and phenotype. PD-L1 + CSCs were positively correlated with Tregs, PD-1 + CD4 + T, and Tim3 + CD4 + T. PD-L1 + CSCs had an immunosuppressive potential, which correlated with LNM, progressed lung cancer, poor prognosis, and drug resistance [105].

The immune microenvironment is also inextricably linked to the inflammatory manifestations of tumors. Immune-infiltrated NSCLC cells had high expression of soluble immune suppressive factors indoleamine 2,3-dioxygenase 1 (IDO1) and PD-1. IDO1 was higher expressed in inflamed tumors and tertiary lymphoid structures (TLS), a robust predictor of immune checkpoint inhibitors (ICI) efficacy. In a T-cell-inflamed TME and/or TLS, targeting IDO pathway combining anti-PD-1/PD-L1, might be a potential target to reinvigorate TLS-driven antitumor immunity [111]. TLS maturation is also associated with major pathological response, and being an independent predictor for DFS in resectable neoadjuvant chemoimmunotherapy (anti-PD-1 antibody plus chemotherapy) [112]. Removal of the mediastinal lymph nodes (MLN) increased the anti-tumoral activity of NK cell and reversed its exhaustion [113]. Meanwhile, MLN removal could improve immunosenescent phenotype, immune checkpoint receptor expression, and cytotoxicity by CTLs [114].

The interference of tumor lymphangiogenesis by macrophage polarization intervene also provides a new perspective on personalized treatment. Mouse macrophages (predominantly infiltrated M2-like TAMs) treated with Paclitaxel (PTX) triggered lymphatic vascular activity in naive tumor cells in a VEGF-C/VEGFR-3-dependent manner, which facilitated LEC invasiveness and migratory properties [115]. Macrophages expressing VEGFR-3 stimulated tumor metastasis by releasing the histone cathepsin, which enhanced the activity of the heparanase. Thus, blocking the VEGF-C/VEGFR3 axis in activated macrophages not only directly inhibited lymphangiogenesis but also blocked the pro-metastatic activity of macrophages in mice after chemotherapy [115]. Studies have shown that high VEGF-C with high M2 ratio (CD163+/CD68+) was an independent prognostic factor in NSCLC patients and significantly associated with angiogenesis and lymphangiogenesis [127].

TGF-β signaling ensured the structure integrity of lymphatic vessels and lymphatic homeostasis, encouraging tumor lymphatic metastasis in LECs [116]. In early LSCC, the expression of TGF-β1 in PDPN + CAFs was higher, which was correlated with CD204 + TAM infiltration, suggesting that it was related to the immunosuppressive microenvironment [117]. As mentioned above, TGF-β1 might work in the process of inducing EMT and promoting the acquisition of CSCs-like characteristics [118]. TGF-β-induced active demethylation could upregulate CD147, targeting CD147 methylation might be beneficial in preventing tumor invasion and metastasis [119].

## Migration and distant metastatic of NSCLC cells

### Epithelial-mesenchymal transition (EMT) and cell migration

LECs might regulate the migration and adhesion of tumor cells in addition to lymphangiogenesis. EMT is an important process in lymphatic dissemination and metastasis. EMT phenotype can be characterized by epithelial markers (E-Cadherin and β-Catenin) and mesenchymal markers (N-Cadherin and Vimentin) [120]. KAI1 inhibited β-Catenin-mediated EMT and played a metastatic inhibitory role during tumor invasion, angiogenesis, and lymphangiogenesis [121]. Downregulation of miR-148a was found to be associated with NSCLC LNM as well as shorter survival, DNMT1 expression was reduced by miR-148a overexpression, which resulted in decreased DNA methylation of E-Cadherin, leading to a rise in E-Cadherin protein levels [122]. In pathological N0 lymph nodes of NSCLC patients, micrometastatic tumor cells as well as positive expression of VEGF-C were analyzed. There was also a relationship between lymphangiogenesis, micrometastasis and adhesion molecules with specific histology (E- Cadherin, α/β/γ- Catenin) [123]. Transcription factors of EMT, SNAI2 and TWIST1, were also involved in lymph node progression, dependent on the TGF-β pathway partly [124, 125].

Inhibition of VEGF-C paracrine inhibited migration, invasion, and EMT in lung cancer cell lines and reduced the percentage of CSCs-like cells, reducing tumor drainage and cancer cell spread [41]. EMT promoted cancer progression by cancer stem cells (CSCs) forming that are more tumorigenic [55]. TGF-β might work in the process of inducing EMT and promoting the acquisition of CSCs-like characteristics, then upregulating VEGFR-3 expression [118]. Another study testified TGF-β exposure enhanced tumor cell adhesion and migration on LECs. In vivo experiments, targeting of TGF-β and integrin β3 significantly reduced LNM, suggesting a more effective combination therapy [126].

JAM-C promoted lymphangiogenesis and LNM by increasing the migratory capacity of cancer cells and modulating VEGF-C-mediated activation of integrinβ1 or ERK [48]. p-S6 was also significantly elevated in NSCLC patients with LNM. Inhibition of p-S6 reduced the migration and invasion of NSCLC cells [127].

### Distant metastasis

We know that lymphangiogenesis is often accompanied by sentinel LNM [128]. While distant metastasis may also be derived from LN metastasis, or immunological tolerance induced by LN colonization. Lymphangiogenic metastatic sites exist in distant LNs or organs. It is extrapolated that the induction of lymphangiogenesis occurs at these metastatic sites, which further accelerates dissemination by transgenic mouse models in other tumors with metastases [11, 129]. Overexpression of VEGF-C in tumor cells encouraged intra-lymphatic spread of metastases. In clinical studies, we have found axillary lymph nodes being a rare site of LNM had a higher incidence of metastasis in BRAF mutated patients with NSCLC compared to those in non-BRAF mutated [130]. While whether the molecular mechanisms of lymphangiogenesis at distant metastasis are the same as the aforementioned need to be further explored in cancer models used in experiments.

## **Anti-lymphangiogenic therapy**

It has long been appreciated that patients bearing LNM suffered poor prognosis compared with those lacking LNM. While the treatment paradigm for NSCLC has changed dramatically in the last decades. For resectable NSCLC, lobectomy with regional LN dissection is the primary treatment. While for locally advanced NSCLC, it is prone to combined with chemotherapy, immunotherapy, targeted therapy or radiotherapy as neoadjuvant therapies [131].

Systematic lymphadenectomy is an crucial component of complete surgical operation for resectable NSCLC [132–134]. In the case of small-sized NSCLC, a comprehensive LN dissection is advised during surgery [135]. However, it has been debatable whether lymphadenectomy is helpful for the overall survival of patients [132, 133]. Measures to improve the quality of LN dissection in NSCLC are still needed to explore in the future. For instance, techniques such as transcervical extended mediastinal lymphadenectomy (TEMLA) could improve the 5-year OS of patients with stage IIIA-IIIB (N2) NSCLC after neoadjuvant chemotherapy or chemoradiotherapy [136, 137]. The role of removing tumor draining LNs helps better understand immunotherapeutic approach.

Research data have showed immunotherapies had considerably improved the survival of patients with NSCLC, even at the end of life [138, 139]. Treating patients harboring PD-L1 + CSCs and PD-1 + CD4 + T cells with anti-PD-1/PD-L1 therapies might improve their prognosis [105]. Neoadjuvant immunotherapy elicited a substantial response in LNM and was more effective in LN downstaging [140]. In clinical trials, neoadjuvant pembrolizumab with chemotherapy followed by resection in resectable and early-stage NSCLC, or neoadjuvant nivolumab plus platinum-based chemotherapy in resectable and stage IIIA or IIIB NSCLC, both enjoyed better prognosis than with chemotherapy alone [141, 142].

Maximum standardized uptake (SUVmax) and mean standardized uptake (SUVmean) values were significantly higher in patients with LNM than in who without LNM before underwent preparative PET/CT for diagnosis and staging [143]. The prognosis of NSCLC patients could be predicted by combining the coefficient of variation (CoV) of 18 F-FDG PET/CT images of the primary tumors with the CoV of the targeted LNs [144]. A significant factor in determining a high degree of locoregional tumor control following definitive chemoradiotherapy, was the high sensitivity detection of involved LNs and their incorporation into the radiation target volume [145]. Combining anti-PD-1 blockade with radiotherapy could overcome immunotherapy resistance and strengthen the immune response. The InTRist study provided the strategy for anti-PD-1 toripalimab plus chemotherapy followed by concurrent chemoradiotherapy (cCRT) for bulky LA-NSCLC [146].

As an antibody targeting VEGF-A, bevacizumab combined with ICIs, with or without chemotherapy had been approved for the initial treatment of unresectable NSCLC [147]. However, studies on NSCLC lymphangiogenesis-specific targeted agents were still incomplete. Numerous studies on the molecular mechanisms of NSCLC lymphatic metastasis demonstrate the potential of lymphangiogenic therapeutic targets. Some drugs might delay the progression of NSCLC by modulating the tumor lymphangiogenic pathway (Fig. 2). Studies emerged on monoclonal antibodies (mAb) targeting VEGF-C/D [148, 149] and VEGFR-2/3 [150, 151], as well as soluble constructs of VEGFR-2/3 [152, 153]. These serve as anti-angiogenic targets and can also provide ideas for anti-lymphangiogenesis. Mice inoculated with Lewis lung cells (LLC) -sVEGFR-2 had significantly fewer LYVE-1 + lymphatic vessels and pulmonary lymph node micrometastases, and VEGFR-2, VEGFR-3, and MMP were inhibited. sVEGFR-2 could be a target for inhibiting the VEGF-C pathway that impedes lymphatic metastasis in NSCLC [154]. EGFR-TKIs (gefitinib, afatinib, and AZD9291) could inhibit VEGF-C secretion, further impairing the proliferation, migration, and tube formation of LECs. The three EGFR-TKIs reduced the expression of VEGFR-2/3, VEGF-C, and CCR7 through the JAK/STAT3 signaling pathway, FAK phosphorylation and c-Myc, this mechanism reduced NSCLC cell proliferation and metastasis to lymphatic vessels to prevent distant metastasis. Meanwhile, afatinib suppressed tumor growth and lymphangiogenesis in a dose-dependent manner in the xenograft mouse model [155]. The small molecule inhibitor Nintedanib blocked the structural domains of all FGFR, PDGFR, and VEGFR receptors by binding to ATP-binding sites in kinases. Animal studies demonstrated a significant reduction in intratumoral LVD treated with Nintedanib. Nintedanib inhibited VEGF-C-induced signaling blocking tumor lymphangiogenesis in NSCLC cells [156]. IL-1/IL-1R signaling driven inflammatory stimuli and upregulation of VEGF-C expression enhanced lymphangiogenesis. Experiments demonstrated that the IL-1R antagonist anakinra inhibited tumor growth, lymphangiogenesis, and LNM by suppressing VEGF-A and VEGF-C expression, and levels of CXC chemokines in macrophages co-cultured with highly metastatic cancer cells [95]. Bioinformatics analysis revealed that high expression of BIRC5/HIF1A/FLT4 was associated with primary NSCLC lymphangiogenesis and metastasis. Small molecule kinase inhibitor drugs have been shown to have better specificity, selectivity, and safety than conventional chemotherapeutic agents. Among them, the quinoline-derived small molecule HN-N07 inhibited the target genes BIRC5/HIF1A/FLT4, and became a potential inhibitor of the NSCLC cancer-causing signaling pathway [157]. Some of the growth factors and their receptors mentioned above, as well as other mechanistic targets, may be potential targets for LN-positive NSCLC. For example, aspirin reduces PDGF and VEGF levels in platelet release [158]. The COX-2-specific inhibitor Celecoxib led to a reduction in COX-2 and VEGF-C expression and LYVE-1-positive vessels [96]. This all requires further clinical trials to confirm the efficacy of pathway target inhibitors in NSCLC lymphatic metastasis.

Furin-like pro-protein convertases (FPPC), strongly upregulating in tumor cells, could cleave signaling protein semaphorin-3 C (sema3C), which induced cytoskeletal collapse in LECs. Whereas active point mutation of furin protease cleavage-resistant sema3C (FR-sema3C) inhibited VEGF-C-induced phosphorylation of VEGFR-3, ERK1/2, and AKT, which inhibited the proliferation of LECs. This suggested that full-length FR-sema3C may be further developed into a novel antitumor drug [159]. Itraconazole (ITCZ) was a potent inhibitor of endothelial cell proliferation and inhibits angiogenesis. It was found that expression of LMVD and VEGF-C were significantly reduced in the high-dose ITCZ group. It suggests that ITCZ may inhibit malignant pleural effusion by inhibiting lymphangiogenesis in mice [160].

Further, endobronchial endotumor chemotherapy was used through systemic radiotherapy or before surgery. Endotumor lymphatic therapy cloud be administered by using a needle catheter through a flexible bronchoscope to inject cisplatin or other cytotoxic agents, into malignant tissue located in the airway lumen or peribranchial structures. Eradication of micrometastases or occult metastases in regional LNs that migrate to the area of the draining tumor was achieved through prophylactic or therapeutic treatment [161]. In addition, siRNAs provide a method to silence specific oncogenes to control tumor growth. Tumor-targeting nanostructured lipid carriers improve the stability, solubility, and cell permeability of drugs and siRNAs. siRNA nanostructures, as an innovative way to meet clinical needs, can be introduced into a variety of therapeutic combinations [162]. The new technology provided new precision for the precise use of drugs for lymphatic vessels.

## Conclusion

It is well known that NSCLC is a disease with poor prognosis, often accompanied by recurrence and metastasis, lymphatic metastasis is a common mode of metastasis for NSCLC. In recent years, researches on neoplastic lymphangiogenesis and LNM in NSCLC have made great progress. The evidence is compelling that the extent of LNM is crucial prognostic judgement for overall survival of patient in NSCLC. Tumor cells and other immune cells secrete cytokines and chemokines, regulating the generation of nascent ducts and promoting the invasion and metastasis in tumor lymphatic microenvironment. Numerous studies have analyzed the correlation between clinicopathological features and lymphatic vessel-related indicators. And there are already some standard quantifiable assays for identifying robustly expressed lymphatic vessel markers. However, currently commonly used markers, such as PDPN or LYVE-1, are also expressed epithelially on other cells or regulated by other factors, which can lead to experimental errors.

This review combed the pathways of NSCLC-regulated lymphatic vessel formation. This will help to understand the specific mechanisms of lymphatic vessel formation in the TME. Genomics, transcriptomics, and proteomics can help identify potential drivers and assist in predicting efficacy and prognosis. Single-cell sequencing techniques can also detect cell population heterogeneity to better understand the biological changes that occur with tumor-associated LNM.

Individualized combination therapies based on targeting and immunotherapy for NSCLC have been updated over the past decades. Drugs targeting angiogenesis have also gradually moved into a mature stage, particularly bevacizumab, have been approved for use in clinical treatment and have achieved good therapeutic effects, so the research of specific drugs targeting lymphangiogenesis has great potential. It is targeting key molecules in the lymphatic immune microenvironment and reversing tumor immunosuppression. By synergizing with immune checkpoint inhibitors and targeting suppressive immune cells to reduce tumor lymphatic vessel formation. In addition, the theory and practice of surgery and radiotherapy have been advancing. Precise and stabilized localizing of pivotal molecules in the lymphangiogenic pathway through si-RNA technology. All of this requires more prospective biomarker trials and extensive clinical trials. Identification of lymphoid markers as well as designing better experimental models to validate theories are all future endeavors. The future holds promise for the rationalization of combining anti-lymphangiogenic drugs with current targeted or immunological approaches.

## Data Availability

No datasets were generated or analysed during the current study.

## Abbreviations

NSCLC: non-small cell lung cancer
LN: lymph node
LECs: lymphatic endothelial cells
ISF: interstitial fluid
LSMCs: lymphatic smooth muscle cells
LV: lymphatic vessel
LVI: lymphatic vessel invasion
TME: tumor microenvironment
TDLN: tumor-draining lymph nodes
PD-1: programmed death 1
PD-L1: programmed death ligand 1
DCs: dendritic cell
TDSFs: tumor-derived secreted factors
EVs: extracellular vesicles
SCS: subcapsular sinuses
VEGF: vascular endothelial growth factor
VEGFR: vascular endothelial growth factor receptor
LYVE-1: lymphatic vessel endothelial hyaluronan receptor-1
SOX18: sex-determining region Y-box 18
COUP-TFII: the chicken ovalbumin upstream promoter transcription factor II
Prox-1: Prospero homeobox-1 protein
NRP-2: Neuropilin-2
PDPN: podoplanin
LVD: Lymphatic vascular density
ptLVD: peritumor LVD
LNM: lymph node metastasis
LUAD: lung adenocarcinoma
LSCC: lung squamous cell carcinoma
PIGF: placental growth factor
Mdig: Mineral dust-induced gene
C/EBP-δ: CCAAT/enhancer binding protein-δ
TGF-β: transforming growth factor-β
EPH: ephrin
ANGPTL2: the adipokine angiopoietin-like protein 2
TAMs: tumor-associated macrophages
PDGFs: Platelet-derived growth factors
HGFs: hepatocyte growth factors
IGFBP7: insulin-like growth factor binding protein 7
FGF2: fibroblast growth factor 2
Shh signaling pathway: sonic hedgehog signaling pathway
miRNA/mi-R: microRNA
OS: overall survival
RFS: relapse-free survival
DSS: disease-specific survival
DFS: disease-free survival
IL: Interleukin
IL-7R: IL-7 receptor
Romo1: reactive oxygen modulator 1
LSP: Lipopolysaccharide
TSLP: Thymic stromal lymphopoietin
HLMs: human lung macrophages
CTL: cytotoxic T-lymphocytes
ICB: immune checkpoint blockade
Treg: regulatory T cells
IDO1: indoleamine 2,3-dioxygenase 1
TLS: tertiary lymphoid structures
ICI: immune checkpoint inhibitors
MLN: mediastinal lymph nodes
PTX: Paclitaxel
EMT: epithelial-mesenchymal transition
CSCs: cancer stem cells
JAM-C: junctional adhesion molecule-C
TEMLA: transcervical extended mediastinal lymphadenectomy
CoV: coefficient of variation
cCRT: concurrent chemoradiotherapy
mAb: monoclonal antibodies
FPPC: furin-like pro-protein convertases
sema3C: semaphorin-3 C
FR-sema3C: furin protease cleavage-resistant sema3C
ITCZ: Itraconazole
HUVECs: human umbilical vein–derived endothelial cells
HLECs: human lymphatic endothelial cells
LLC: Lewis lung cells
3LL: Lewis lung adenocarcinoma cells
